# New Coumarin Derivative as an Eco-Friendly Inhibitor of Corrosion of Mild Steel in Acid Medium

**DOI:** 10.3390/molecules20010366

**Published:** 2014-12-29

**Authors:** Ahmed A. Al-Amiery, Yasameen K. Al-Majedy, Abdul Amir H. Kadhum, Abu Bakar Mohamad

**Affiliations:** 1 Department of Chemical and Process Engineering, University Kebangsaan Malaysia (UKM), Bangi, Selangor 43000, Malaysia; E-Mails: dr.jasmin79@gmail.com (Y.K.A.-M.); amir@eng.ukm.my (A.A.H.K.); 2 Environmental Research Center, University of Technology (UOT), Baghdad 10001, Iraq; 3 Fuel cell Institute, Universiti Kebangsaan Malaysia (UKM), Bangi, Selangor 43000, Malaysia; E-Mail: drab@eng.ukm.my

**Keywords:** 4-hydroxycoumarin, ^13^C-NMR, corrosion inhibitor, weight loss

## Abstract

The anticorrosion ability of a synthesized coumarin, namely 2-(coumarin-4-yloxy)acetohydrazide (EFCI), for mild steel (MS) in 1 M hydrochloric acid solution has been studied using a weight loss method. The effect of temperature on the corrosion rate was investigated, and some thermodynamic parameters were calculated. The results indicated that inhibition efficiencies were enhanced with an increase in concentration of inhibitor and decreased with a rise in temperature. The IE value reaches 94.7% at the highest used concentration of the new eco-friendly inhibitor. The adsorption of inhibitor on MS surface was found to obey a Langmuir adsorption isotherm. Scanning electron microscopy (SEM) was performed on inhibited and uninhibited mild steel samples to characterize the surface. The Density Function theory (DFT) was employed for quantum-chemical calculations such as E_HOMO_ (highest occupied molecular orbital energy), E_LUMO_ (lowest unoccupied molecular orbital energy) and μ (dipole moment), and the obtained results were found to be consistent with the experimental findings. The synthesized inhibitor was characterized by Fourier transform infrared (FTIR) and nuclear magnetic resonance (NMR) spectroscopic studies.

## 1. Introduction

Mild steel (MS) finds use in extensive industrial applications such as handling of acids, alkalis, and salt solutions. The aggressiveness of these substances causes severe corrosion to engineering structures made of mild steel, which leads to huge financial and material losses. Hence, the study of mild steel corrosion and the inhibition of mild steel corrosion of have invited the attention of scientists and technocrats to devise ways to control the corrosion. Among the various corrosion control measures, the use of corrosion inhibitors is a familiar method. It is known that corrosion inhibitors act by adsorbing on the metal surface [1,2]. Several chemical compounds have been tested to date as corrosion inhibition of metals and alloys, and compounds having in their structures *N*, *S*, and *O* hetero-atoms, incorporated in an aromatic system, have been found to possess excellent anticorrosion potential. In recent years, due to environmental issues, researchers have been working on the concept of negligible harmful effects to the environment (green inhibitors) to avoid the toxic effect of synthetic corrosion inhibitors. This new class of inhibitors is found to be highly efficient in acidic media. For the same purpose, various plant extracts have also been studied to control the corrosion of metals in acidic media [3,4,5].

The use of environmental friendly corrosion inhibitors is nowadays very common because they are cost effective and eco-friendly [6,7,8,9,10,11]. To this end, the use of organic compounds containing nitrogen, oxygen, and/or sulfur in a conjugated system as inhibitors to reduce corrosion attack has received detailed attention [12,13,14,15]. In this work a new green corrosion inhibitor derivative from 4-hydroxycoumarin was successfully synthesized and fully characterized by infra-red (IR) and nuclear magnetic resonance (NMR) spectroscopic studies, in addition to micro elemental analysis CHN. Weight loss tests were applied to test the inhibitory properties of the synthesized compound in carbon steel immersed in 1.0 M HCl. The new inhibitor showed inhibitory properties dependent on oxygen and nitrogen atoms. The highest efficiency was confirmed by scanning electron microscopy.

## 2. Results and Discussion

### 2.1. Chemistry

**Scheme 1:**
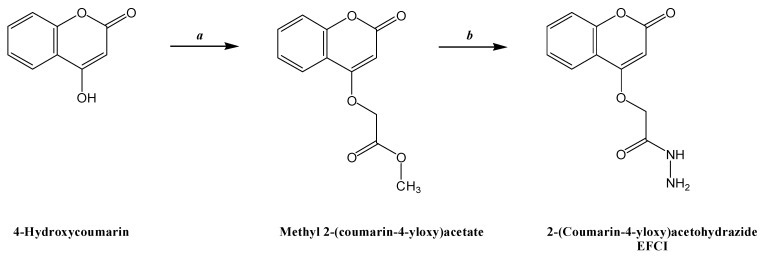
Synthesis of the corrosion inhibitor EFCI.

The reaction sequence for the synthesis of the new green inhibitor derived from 4-hydroxycoumarin is outlined in Scheme 1. Methyl 2-(coumarin-4-yloxy)acetate was obtained by refluxing methyl bromoacetate with 4-hydroxycoumarin in anhydrous acetone in the presence of anhydrous potassium carbonate. The FT-IR spectrum of this compound showed an absorption band at 1723.1 cm^−1^ (ester C=O carbonyl stretching). The ^1^H-NMR spectrum exhibited a singlet at δ 3.63 ppm due to the three CH_3_ protons. The reaction of methyl 2-(coumarin-4-yloxy)acetate with hydrazine hydrate afforded the hydrazide EFCI in good yield. The FT-IR spectrum of the compound showed absorption bands at 3233.3 and 3210.0 cm^−1^ (hydrazide NH-NH_2_). The ^1^H-NMR spectrum exhibited a singlet at δ 4.45 ppm due to the two CH_2_ protons and a singlet due to the single NH proton at δ 8.21 ppm. The ^13^C-NMR spectrum exhibited a doublet at 36.92 and 37.38 ppm due to the CH_2_ carbon and a singlet due to the single CH_3_ carbon at 29.72.

### 2.2. Weight Loss Method

#### 2.2.1. Effect of *Concentration*

**Figure 1:**
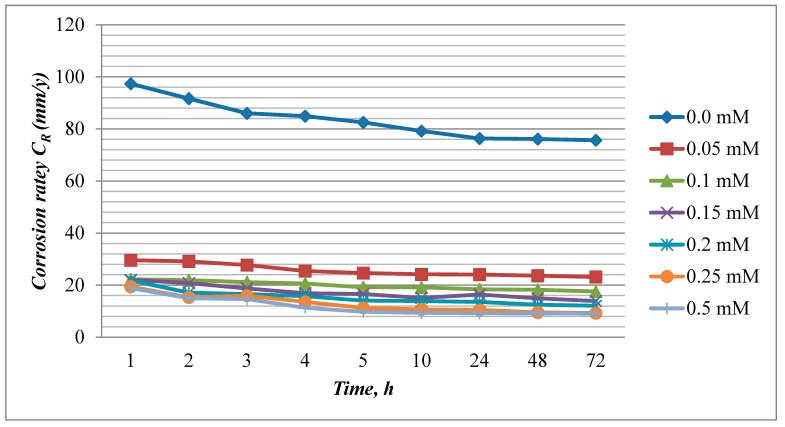
Influence of concentration of EFCI and time on corrosion rate of mild steel at 303 K.

**Figure 2:**
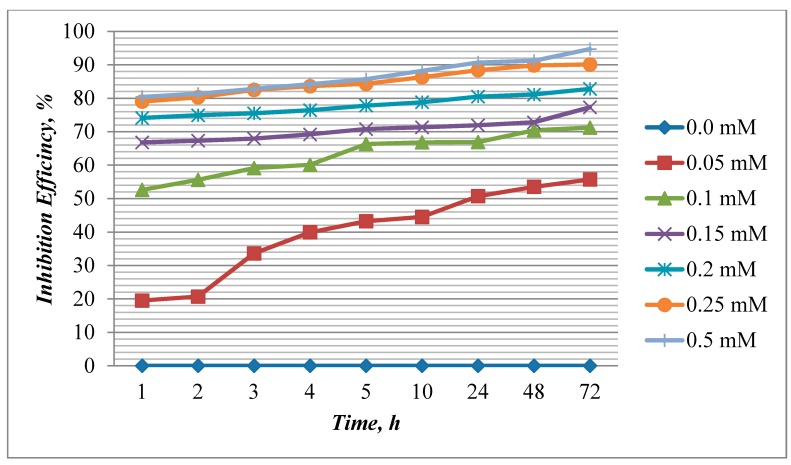
Influence of concentration of EFCI and time on inhibition efficiency of mild steel at 303 K.

The inhibition efficiency and corrosion rate values calculated from weight loss measurements for mild steel in acid media with various concentrations of EFCI for a period of time (1, 2, 3, 4, 5, 10, 24 48 and 72 h), at 303 K are shown in Figure 1 and Figure 2. EFCI markedly reduced the corrosion of mild steel in acid media. The inhibition efficiency increased with a rise in concentration of the green inhibitor and reached a maximum IE(%) at 0.5 mM concentration of EFCI. The increase in IE(%) with the increase in concentration is suggestive of the increase in the extent of protection efficiency of EFCI.

#### 2.2.2. Effect of Temperature

**Figure 3:**
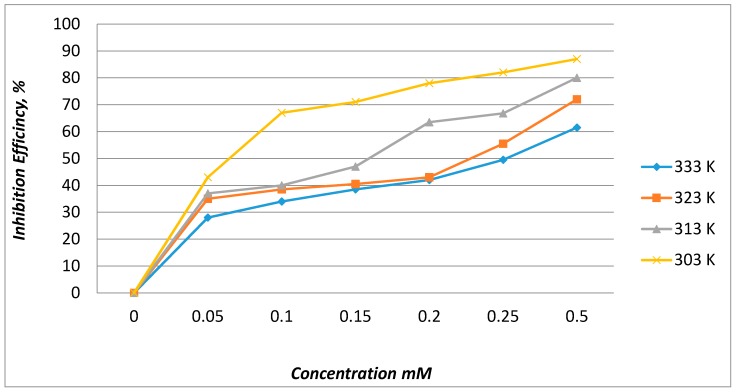
Effect of temperature on inhibition efficiency of EFCI with various concentrations.

A comparison of the inhibition efficiency of EFCI on MS in acid solutions in the absence and presence of various concentrations of EFCI at various temperatures (303, 313, 323 and 333 K) indicated that IE enhanced was with an increase in inhibitor concentration and decreased with an increase in temperature (Figure 3). In the adsorption process of organic compounds, the heat of adsorption is generally negative, and this indicated an exothermic process. This is the reason that the inhibitor efficiency decreases at a higher temperature.

### 2.3. Scanning Electron Microscopy (SEM) Analysis

**Figure 4:**
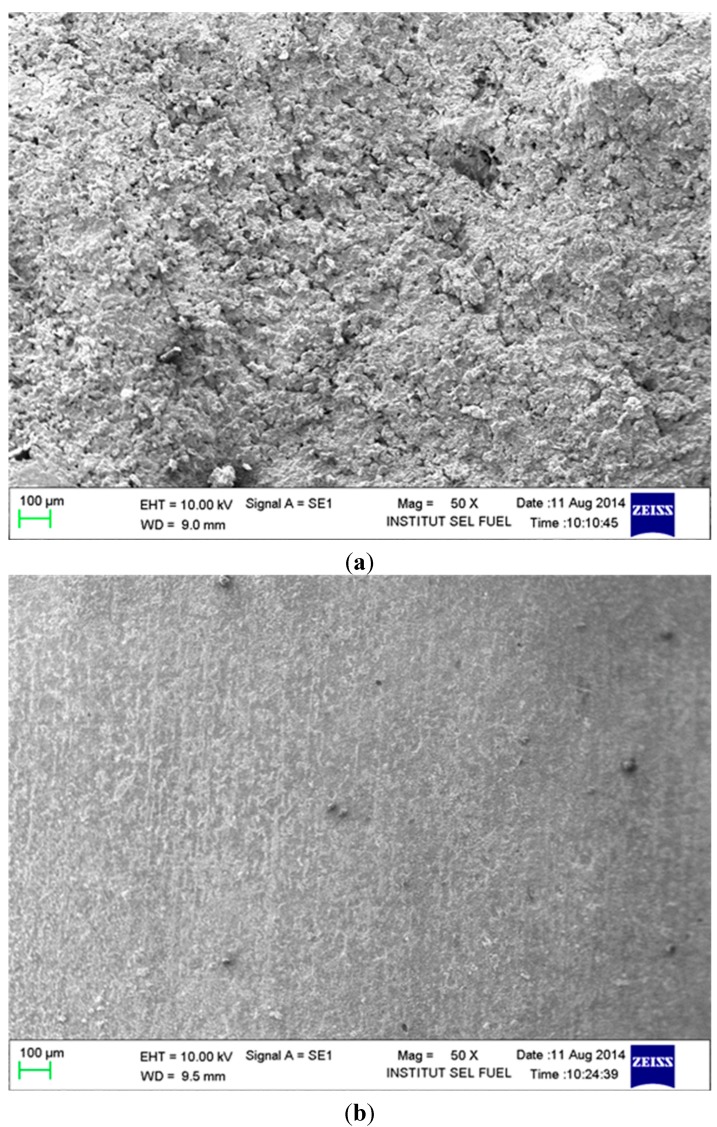
The SEM micrographs 5000×, for mild steel in 1.0 M HCl with 0.5 mM of the corrosion inhibitor at 30 °C for 5 h as immersion time. (**a**) absence the inhibitor; (**b**) in presence the inhibitor.

Based on Figure 4a, as expected, the mild steel surface, which was originally clean and smooth, suffered from serious corrosion and became rough. The mild steel surface was significantly attacked by HCl. Based on Figure 4b, the treated mild steel surface did not suffer serious corrosion. The corrosion inhibitor thus provided protection to the mild steel from the corrosion attack caused by HCl.

### 2.4. Adsorption Isotherm

Corrosion inhibitors act by adsorption of ions or molecules over metal surfaces. They reduce the corrosion rate mainly by increasing or decreasing the anodic and/or cathodic reactions, decreasing the diffusion rate for reactants to the surface of the metal and the electrical resistance of the metal surface [16]. Adsorption depends mainly on the charge and the nature of the metal surface, electronic characteristics of the metal surface, adsorption of solvents and other ionic species, on the electrochemical potential at solution interface. The adsorption mechanism of organic corrosion inhibitors on metal surface can be explained via the study of adsorption isotherm and adsorptive behavior of the inhibitor. The most frequently used adsorption isotherms are the Langmuir, Temkin, Frumkin, and Freundluich isotherms [17]. The corrosion inhibition of organic inhibitors on mild steel in hydrochloric acid can be described by a molecular adsorption method.

The adsorption process is influenced by the structure of the organic compounds, the charge distribution in the molecules, the nature of the surface-charged metals and the types of media used [18,19]. The values of surface coverage, (where θ is the surface coverage) for the different concentrations of the studied inhibitors have been used to explain the best adsorption isotherm to determine the adsorption process. To calculate the surface coverage θ, it was assumed [20] that the IE (%) is due mainly to the blocking effect of the adsorbed species and hence Equation (1) applies:

(1) $$\text{θ}=\frac{\text{IE \%}}{100}$$

In this work, the surface coverage θ was calculated from the above relation using the IE calculated from the weight loss technique. The plots of $\frac{C_{inh}}{\theta }$ against $C_{inh}$ yield a straight line with an approximately unit slope, indicating that the inhibitor under study obeys the Langmuir adsorption isotherm [21], as in the Equation (2):

(2) $$\frac{C_{inh}}{\theta }=\;\frac{1}{K_{ads}}+\;C_{inh}$$

where $C_{inh}$ is the concentration of the inhibitor and $K_{ads}$ is the adsorption constant obtained from the intercept of the straight line. $K_{ads}$is associated with the standard free energy of adsorption $∆G_{ads}^{o}$.

$∆G_{ads}^{o}$ is given by Equation (3):

(3) $$∆G_{ads}^{o}=\;-RTln\left[55.5\;K_{ads}\right]$$

where the value of 55.5 represents the molar concentration of water in solution expressed in units of M. R is the universal gas constant and T is the absolute temperature.

**Figure 5:**
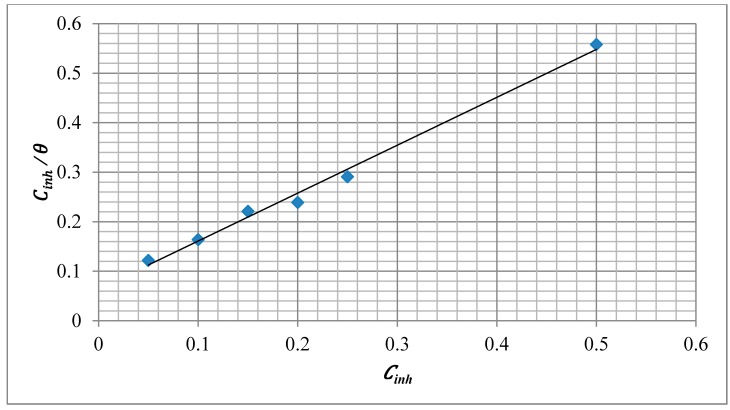
Adsorption isotherm for mild steel in 1.0 M HCl with different concentrations of the corrosion inhibitor.

The value of *K_ads_* and $∆G_{ads}^{o}$ was calculated according to Figure 5, and the estimated $∆G_{ads}^{o}$ was −26.15 kJ/mol. The negative value of $∆G_{ads}^{o}$ indicates a spontaneous adsorption of the inhibitor on the mild steel surface and a strong interaction between the inhibitor molecules and the surface of the mild steel. Generally, a value of $∆G_{ads}^{o}$ around −20 kJ/mol is consistent with physical adsorption, while a value of $∆G_{ads}^{o}$ around −40 kJ/mol or higher is chemical adsorption occurring with the sharing or transfer of electrons from organic molecules to the surface of the mild steel.

### 2.5. Corrosion Kinetic Parameters

Arrhenius and transition state plots were used for determination of $E_{a}$ (activation energy), $∆H_{a}$ (activation enthalpy) and $∆S_{a}$ (activation entropy) at the studied temperature 303, 313, 323 and 333 K for the mild steel in the presence and absence of EFCI (Equation (4)):

(4) $$C_{R}=Ae^{-E_{a}/RT}$$

where $C_{R}$ is the corrosion rate, A is pre-exponential factor, $E_{a}$ (J·mol^−1^) and R is gas constant (8.314 J·mol^−1^·K^−1^).

To solve this equation, we take natural logs of both sides (common logs could be used as well):

(5) $$lnC_{R}=\left[\frac{-E_{a}}{RT}\right]+lnA$$

**Figure 6:**
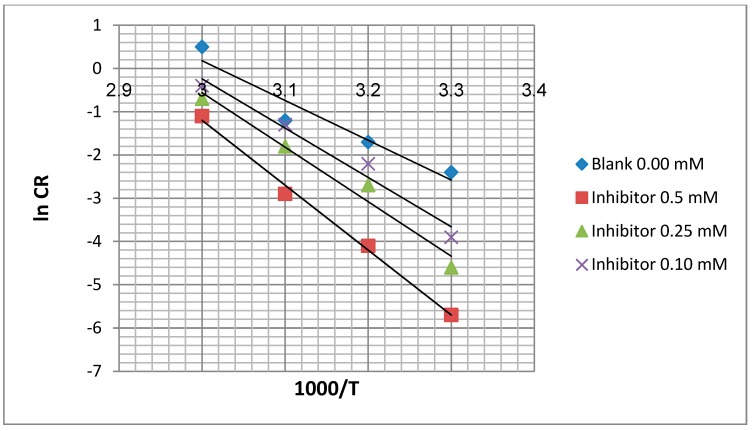
Arrhenius plot for mild steel in 1.0 M HCl.

The values of *E_a_* were calculated (Table 1) from the slope (slope = −*E_a_*/*R*) of the straight line of the x-axis (ln *C_R_*) and y-axis (1000/*T*) of the graph by using of Arrhenius plot for the mild steel in 1.0 M HCl in the with and without EFCI (Figure 6).

**Table 1:** Corrosion kinetic parameters for mild steel in 1.0 M HCl in the presence and absence of EFCI.

Concentration	*E_a_* (kJ·mol^−1^)	ΔH_a_ (kJ·mol^−1^)	ΔS_a_ (J·mol^−1^·K^−1^)
Blank	61.75	59.22	−70.53
0.10 mM	80.85	79.15	14.11
0.25 mM	90.0	87.15	17.74
0.5 mM	94.12	92.36	22.27

The transition state equation was used to calculate the Δ*H*_a_ and Δ*S*_a_:

(6) $$C_{R}=\frac{RT}{hN}exp\left(\frac{∆S_{a}}{R}\right)exp\left(\frac{-∆H_{a}}{RT}\right)$$

Where *N* is Avogadro’s number (6.02 × 1023 mol^−s^) and *h* is Plank’s constant (6.63 × 10^−s^·m^2^·kg·s^−k^).

To carry out simple calculations, Equation (7) was rearranged to become:

(7) $$ln\left[\frac{C_{R}}{T}\right]=-\left[\frac{∆H_{a}}{RT}\right]+\left\{ln\left[\frac{R}{hN}\right]+\left[\frac{∆S_{a}}{R}\right]\right\}$$

**Figure 7:**
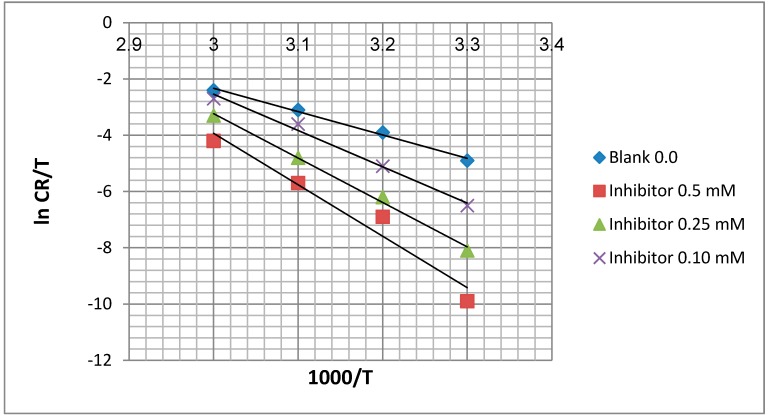
Transition state plots for mild steel in 1.0 M HCl.

A plot of ln (*C_R_*/*T*) against 1000/*T* gives a straight line with the slope equal to (−Δ*H*a/*R*) and intercept equal to [ln(*R*/*Nh*) + (Δ*S*_a_/*R*)], as shown in Figure 7. The Δ*H*_a_ and Δ*S*_a_ values were calculated and are displayed in Table 1.

From Table 1, the activation energy increases in the presence of the inhibitor, implying that a physical adsorption (electrostatic) process occurred in the initial stage. In addition, the *E_a_* values are greater than 20 kJ·mol^−1^ in both the presence and absence of the inhibitor, which indicate that the entire process is controlled by the surface reaction [22]. According to Szauer and Brand, the increase in activation energy can be attributed to the decrease in the adsorption of the inhibitor on the mild steel surface with increases in temperature [23,24]. The values of *E_a_* and Δ*H_a_* are higher in the presence of the inhibitor. This result shows that the energy barrier of the corrosion reaction is increased without changing the mechanism of dissolution. The endothermic nature of steel dissolution is indicated by the positive values of Δ*H_a_* for both the corrosion processes with and without the inhibitor.

Meanwhile, the positive values of Δ*S_a_* reveal that the adsorption process is accompanied by a increase in the entropy which acts as a driving force for adsorption of the inhibitor on the mild steel surface. The value of Δ*S_a_* increases in the presence of the inhibitor and is generally interpreted by increases in the disorder, as the reactants are converted to activated complexes [25].

### 2.6. Suggested Mechanisms of Actions of New Synthesized Compound as Inhibitor

**Scheme 2:**
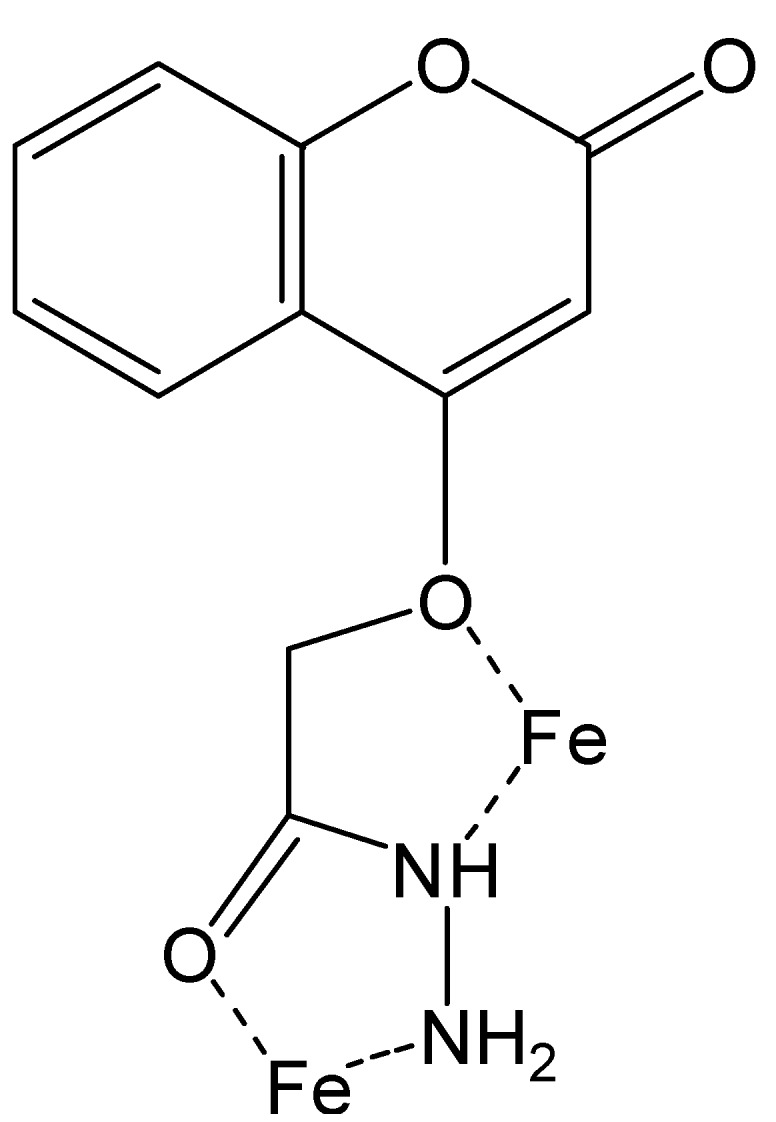
Synthesis of the corrosion inhibitor EFCI.

Chemically the inhibitor is adsorbed on the metal surface and forms a protective thin film or chemical bonds form by reaction between the inhibitor and metal. The adsorption mechanism of organic inhibitors can proceed via one of these routes. 1st, charged molecules and metal attract electrostatically. 2nd, the interaction between unpaired electrons and the metal surface. 3rd, interaction between π-electrons and the metal surface. Organic inhibitors protect the metal surface by blocking cathodic or anodic reactions or both and forming insoluble complexes. The inhibition efficiency of our corrosion inhibitor against the corrosion of mild steel in 1M hydrochloric acid can be explained according to the number of adsorption sites, charge density, molecular size, mode of interaction with the metal surface and ability of formation of metallic insoluble complex. The π electrons for the double bonds and free electrons on the oxygen and nitrogen atoms form chemical bonds with the metal surface as shown in Scheme 2.

### 2.7. Computational Studies

#### 2.7.1. Geometrical Isomers of the EFCI

**Figure 8:**
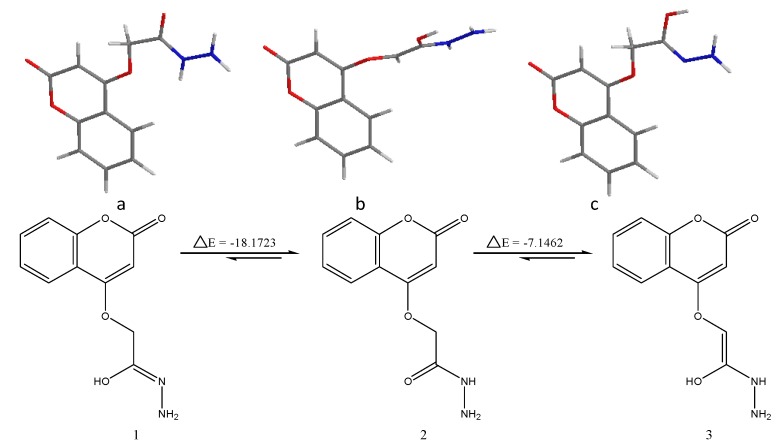
Probable conformational isomers of the *N*-acylhydrazone of EFCI (3-dimensional structures (a, b, c) and geometrical isomers (1, 2, 3)).

With respect to the C=O double bond, *N*-acylhydrazones (NAHs) may exist as *enol or keto* geometrical isomers. Energy calculations performed were done according to the density functional theory (DFT) B3LYP method using the 6–31 G basis set by means of the Gaussian 09, revision A.02 method. These calculationsindicated a slight difference in energy (ΔE = −18.1723 Kcal/mol and −7.1452 Kcal/mol) respectively, between the *enol* and *keto* conformers in the favor of the former (Figure 8). Therefore, we concluded that the EFCI was obtained as single *Z-enol* geometrical isomer.

#### 2.7.2. Quantum Chemical Calculations

**Figure 9:**
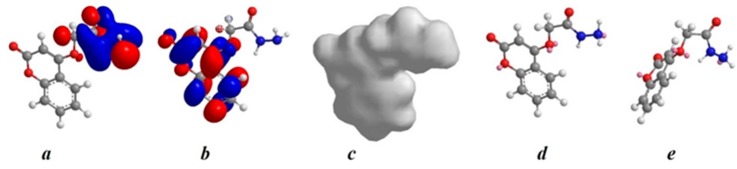
Electronic properties of EFCI (**a**) HOMO orbital; (**b**) LUMO orbital; (**c**) total electron density; (**d**) Fukui (f^−^) function; (**e**) Fukui (f^+^) function.

The structural nature of the organic corrosion inhibitor and inhibition mechanism can be described by Density Functional Theory (DFT). This technique has been found to be successful in providing insights into the chemical reactivity and selectivity in terms of global parameters such as electro-negativity (v), hardness (g) and softness (S), and local softness (sđ~r Þ) [26,27]. The design of the EFCI compound for use as a corrosion inhibitor was based on several factors. First, the molecule contains oxygen and nitrogen atoms as active centers. Second, EFCI can be easily synthesized and characterized [28]. Excellent corrosion inhibitors are usually organic compounds that not only offer electrons to unoccupied orbitals of the metal but also accept free electrons from the metal [29]. Quantum chemical theoretically calculations were used to investigated the interactions between metal and inhibitor [30]. Highest occupied molecular orbital (HOMO), lowest unoccupiedmolecular orbital (LUMO), and Fukui functions as well as the total electron density of EFCI are presented in Figure 9. The blue and red iso-surfaces depict the electron density difference; the blue regions show electron accumulation while the red regions show electron loss. Quantum parameters such as E_HOMO_, E_HOMO_ and Dipole Moment are provided in Table 2. The HOMO regions for the molecule, which are the sites at which electrophiles attack and represent the active centers with the utmost ability to interact with the metal surface atoms, has contributions from carbonyl, amide and amine. On the other hand, the LUMO orbital can accept electrons from the metal using anti-bonding orbitals to form feedback bonds are saturated around the coumarin ring [31]. Correspondingly, a high value of the HOMO energy (E_HOMO_) indicates the tendency of a molecule to donate electrons to an appropriate acceptormolecule with low energy or an empty electron orbital, whereas the energy of the LUMO characterizes the susceptibility of molecule toward nucleophilic attack [32]. Low values of the energy of the gap ΔE = ELUMO−EHOMO implies that the energy to remove an electron from the last occupied orbital will be minimized, corresponding to improved inhibition efficiencies [33,34]. E_HOMO_ value (Table 2) do not vary very significantly for EFCI, which means that any observed differences in the adsorption strengths would result from molecular size parameters rather than electronic structure parameters. The seemingly high value of ΔE is in accordance with the nonspecific nature of the interactions of the molecule with the metal surface. A relationship between the corrosion inhibition efficiency of the EFCI with the orbital energies of the HOMO (E_HOMO_) and LUMO (E_LUMO_) as well as the dipole moment (μ) is shown in Table 2. As is clearly observed, the inhibition efficiency increases with an increase in E_HOMO_ values along with a decrease in E_LUMO_ values. The increasing values of E_HOMO_ indicate a higher tendency for the donation of electrons to the molecule with an unoccupied orbital. Increasing values of E_HOMO_ thus facilitate the adsorption of the inhibitor. Thus, enhancing the transport process through the adsorbed layer would improve the inhibition effectiveness of the inhibitor. This finding can be explained as follows. E_LUMO_ indicates the ability of the molecule to accept electrons; therefore, a lower value of E_LUMO_ more clearly indicates that the molecule would accept electrons [35]. The direction of a corrosion inhibition process can be predicted according to the dipole moment (μ). Dipole moment is the measure of polarity in a bond and is related to the distribution of electrons in a molecule. In spite of the fact that literature is conflicting on the utilization of μ as an indicator of the direction of a corrosion inhibition reaction, it is for the most part concurred that the adsorption of polar compounds having high dipole moments on the metal surface ought to prompt better inhibition efficiency. The data obtained from the present study indicate that the EFCI inhibitor has the value of μ = 1.394 and highest inhibition efficiency (94.7%). The dipole moment is another indicator of the electronic distribution within a molecule. A few researchers express that the inhibition efficiency increments with increasing values of the dipole moment, which relies on upon the sort and nature of molecules considered. However, there is a lack of agreement in the literature on the correlation between μ and IE %, as in some cases no significant relationship between these values has been identified [36,37].

**Table 2:** Calculated Quantum Chemical Properties for the Most Stable Conformation of EFCI.

Function	Values
E_HOMO_	−0.3766 Hartree
E_LUMO_	−0.1383 Hartree
E_HOMO_– E_LUMO_	−0.2382 Hartree
$f_{\text{max}}^{-}$	0.125
$f_{\text{max}}^{+}$	0.091
Dipole Moment	1.394

The electron density (charge distribution) is saturated all around molecule; hence we should expect flat-lying adsorption orientations [33]. The local reactivity of molecule was analyzed by means of the Fukui indices (FI) to assess reactive regions in terms of nucleophilic (f^+^) and electrophilic (f^−^) behavior. Figure 9d shows that the f^−^ functions of molecule correspond with the HOMO locations, indicating the sites through which the molecule could be adsorbed on the metal surface, whereas f^+^ (Figure 7e) correspond with the LUMO locations, showing sites through which the molecule could interact with the nonbonding electrons in the metal. High f^−^ values are associated with the *N* and *O* (oxygen for the side chain and for the pyrone ring) atoms functions molecule, whereas *C* atoms of the benzene ring functions possess high f^+^ values.

#### 2.7.3. Mulliken Charge

The Mulliken charge distribution of EFCI is presented in Table 3. Atom can be easily donates its electron to the empty orbital of the metal if the Mulliken charges of the adsorbed center become more negative [38]. It could be readily observed that nitrogen, oxygen and some carbon atoms have high charge densities. The regions of highest electron density are generally the sites to which electrophiles can attach [39]. Therefore, N, O and some C atoms are the active centers, which have the strongest ability to bond to the metal surface. Conversely, some carbon atoms carry positive charges, which are often sites where nucleophiles can attach. Therefore, EFCI can also accept electrons from Fe through these atoms. It has been reported that excellent corrosion inhibitors can not only offer electrons to unoccupied orbitals of the metal but also accept free electrons from the metal [40]. According to the description of frontier orbital theory, HOMO (Figure 9) is often associated with the electron donating ability of an inhibitor molecule. The molecules have tendency to donate electrons to a metal with empty molecule orbital if they have high E_HOMO_ values. E_LUMO_, conversely, indicates the ability of the molecule to accept electrons [41]. Acceptance of electrons from a metal surface is easier when the molecule has lower value of E_LUMO_ [42]. The gap between the LUMO and HOMO energy levels of inhibitor molecules is another important parameter. Low absolute values of the energy band gap (E = E_LUMO_ − E_HOMO_) mean good inhibition efficiency [43].

**Table 3:** Charges (Mulliken Charges) for the EFCI.

Atoms	Charges	Atoms	Charges	Atoms	Charges	Atoms	Charges
C(1)	0.3299	O(7)	−0.2428	C(13)	0.0309	H(19)	0.0378
C(2)	0.1828	C(8)	0.3929	C(14)	−0.1016	H(20)	0.2210
O(3)	−0.3630	O(9)	−0.3041	C(15)	0.0003	H(21)	0.0947
N(4)	−0.3450	C(10)	−0.2617	C(16)	−0.0850	H(22)	0.0994
N(5)	−0.0837	C(11)	0.2436	C(17)	0.1646	H(23)	0.0994
O(6)	−0.2851	C(12)	−0.1576	H(18)	0.0333	H(24)	0.0697

## 3. Experimental Section

### 3.1. Chemistry

#### 3.1.1. General Information

The chemicals used during synthesis were supplied by Sigma-Aldrich (Selangor, Malaysia). The IR spectra were obtained on a Nicolet 6700 FT-IR spectrophotometer (Thermo Nicolet Corp., Madison, WI, USA), and the values are expressed in cm^−1^. Nuclear magnetic resonance (NMR) spectra were recorded using an AVANCE III 600 MHz spectrometer (Bruker, Billerica, MA, USA), using DMSO as an internal standard and the values are expressed in δ ppm.

#### 3.1.2. Synthesis of Methyl 2-(coumarin-4-yloxy)acetate

A suspension of 4-hydroxycoumarin (0.999 g, 6.17 mmol) in acetone (30 mL) was refluxed with methyl bromoacetate (9.15 mmol) and K_2_CO_3_ (4.69 g, 33.91 mmol) for 12 h. After cooling, the mixture was evaporated to dryness and the residue was partitioned between CHCl_3_ (50 mL) and water (50 mL). The organic phase was dried using Na_2_SO_4_, filtered and evaporated to dryness. The residue was recrystallized from acetone; yield 85%; m.p. 84–85 °C; ^1^H-NMR (CDCl_3_): δ 3.6 (s, 3H, CH_3_), 4.79 (s, 2H, CH_2_) and 5.58 (s, 1H, -C=C-H), 7.3111, 7.555, 7.896 (three s, 1H each, aromatic ring); IR (cm^−1^): 2960 (C-H, aliphatic), 3083.4 (C-H, aromatic), 1760.3 (C=O, ester), 1723.1 (C=O, lactone), 1624.5 (C=C, alkene), 1567.2 (C=C, aromatic).

#### 3.1.3. Synthesis of 2-(Coumarin-4-yloxy)acetohydrazide

A solution of Methyl 2-(coumarin-4-yloxy)acetate (2.34 g, 10 mmol) in ethanol (25 mL) was refluxed with hydrazine hydrate (15 mmol) for 4 h. After concentrating the reaction mixture an oily mass separated out and was recrystallized using ethanol, yield 55%; ^1^H-NMR (CDCl_3_): δ 4.45 (s, 2H, CH_2_), 4.75 (s, 2H, NH_2_), 5.43 (s, 1H, -C=C-H), 7.41–7.78 (m, 4H, aromatic ring), 8.21 (s, 1H, NH); IR (cm^−1^): 3233.3, 3210 (N-H), 2959.0 (C-H, aliphatic), 3083.9 (C-H, aromatic), 1721.4 (C=O, lactone), 1624.2 (C=O, amide).^13^C-NMR (CDCl_3_): δ (ppm) 155.26 (C=O); 154.38 (C=O, lactone) 117.22 and 117.57, d119.35 and 119.85, 131.17, 132.28 (C-aromatic); 40.45 (C-H); d36.92 and 37.38 (–CH_2_); 29.72 (–CH_3_).

### 3.2. Gravimetric Experiments

#### 3.2.1. Mild Steel Specimens

Mild steel specimens obtained from the Metal Samples Company (Saint Marys, PA, USA) were used throughout this study. The composition (wt%) of the mild steel was as follows: Fe, 99.21; C, 0.21; Si, 0.38; P, 0.09; S, 0.05; Mn, 0.05; and Al, 0.01. The specimens were cleaned according to the ASTM standard procedure G1-03 [44]. The measurements were conducted in aerated, non-stirred 1.0 M HCl solutions containing different concentrations of the eco-friendly synthesized compound as green inhibitor.

#### 3.2.2. Weight Loss Method

The mild steel specimens used had a rectangular shape of (2.5 cm × 2.0 cm × 0.025 cm). The specimens were suspended in duplicate in 200 mL of the test solution, with and without different concentrations (0.0, 0.05, 0.1, 0.15, 0.20, 0.25 and 0.50 mM) of the ECFI. After 1, 2, 3, 4, 5, 10, 24, 48 and 72 h of immersion time, the specimens were taken out, washed, dried, and weighed accurately. The inhibition efficiency IE (%) was determined by using Equation (8):

(8) $$\text{Inhibition Efficiency }\left(\text{IE \%}\right)=\left[1-\frac{{\text{w}}_{2}}{{\text{w}}_{1}}\right]\text{ x }100$$

where w_1_ and w_2_ are weight loss of the mild steel without and with the Eco-Friendly Corrosion Inhibitor, respectively. The corrosion rate (CR) was determined by using Equation (9) [45,46]:

(9) $${\text{C}}_{\text{R}}\left(\text{mm}/\text{y}\right)=\frac{87.6\text{W}}{\text{atρ}}$$

where w is the weight loss, ρ is the density of mild steel, a is the area of specimen and t is the time of immersion.

## 4. Conclusions

The results of the present study revealed that the new coumarin derivative 2-(coumarin-4-yloxy)acetohydrazide functioned as a good corrosion inhibitor for MS in 1 M HCl solution in a concentration-dependent mode. IE of new corrosion inhibitor maximum inhibition efficiency was up to 94.7% at 0.5 mM inhibitor concentration, and decreases with a rise in temperature, which is suggestive of physisorption. The new corrosion inhibitor is adsorbed over a MS surface obeying the Langmuir isotherm. The new inhibitor is proved as an efficient organic inhibitor having good inhibitive properties due to presence of nitrogen and oxygen atoms. SEM measurements supported the formation of a protective layer by new corrosion inhibitor on the MS surface. The anticorrosion study of the new corrosion inhibitor clearly revealed its role in the protection of mild steel in acid media.
